# Genovariation Study of Hantavirus in Main Endemic Areas of Hemorrhagic Fever with Renal Syndrome in Hebei Province, China

**DOI:** 10.1371/journal.pone.0159731

**Published:** 2016-07-21

**Authors:** Qi Li, Yanan Cai, Yamei Wei, Xu Han, Zhanying Han, Yanbo Zhang, Shunxiang Qi, Yonggang Xu

**Affiliations:** Institute for Viral Disease Prevention and Control, Hebei Province Centre for Disease Prevention and Control, Shijiazhuang, Hebei Province, China; University of Texas Medical Branch, UNITED STATES

## Abstract

**Background:**

Hemorrhagic fever with renal syndrome (HFRS) is an important infectious disease in Hebei Province. At present, cases from the northeast regions of the province account for >80% of the total incidences. However, studies that examine the region-specific genetic variations of the Hantavirus (HV), the causative pathogen for HFRS, have been lacking.

**Methods:**

Rodents were collected in northeast Hebei Province from 2004 to 2013, and the HV strains used in this study were isolated in 1993. Lung tissues were isolated from the rodents and HV antigen was detected by indirect immunofluorescence. The M1 and M2 fragments of HV *M* region were amplified by reverse transcription polymerase chain reaction (RT-PCR), cloned into pMDl9-T vector, sequenced and compared with representative standard stains for homology and phylogenetic analysis.

**Result:**

A total of 21 samples of HV antigen-positive were collected. Real-time PCR analysis revealed that the 19 rodent lungs and two HV strains were positive for the SEO virus. 11 samples were chosen to sequence, and they shared 95.8%–99.8% in nucleotide homology, and 83.6%–99.2% when compared to the standard strains of SEO virus. Phylogenetic analysis demonstrated that all strains were grouped into the same S3 subtype.

**Conclusion:**

SEO was the major epidemic genotype of HV in the main HFRS endemic areas in Hebei Province, and S3 was the major subtype. There was minor genetic variation in HV over short term periods, while long term variations were higher.

## Introduction

Hemorrhagic fever with renal syndrome (HFRS), a rodent-borne disease caused by different hantaviruses from family *Bunyaviridae* [1,2,3], is characterized by fever, acute renal dysfunction, and hemorrhage manifestations[4,5]. China is the most severe endemic country of HFRS in the world with 40,000–60,000 cases reported annually, which accounts for more than 90% of the total number of cases worldwide [6]. At present, HFRS is endemic in all 31 provinces of China, where it is a significant public health problem that threatens people’s livelihoods.

Hantaviruses (HV) are rodent-borne members of the family *Bunyaviridae*. HVs are negative-sense, single-stranded RNA viruses possessing a tripartite genome, consisting of large (*L*), medium (*M*) and small (*S*) segments encoding a viral RNA polymerase, envelope glycoproteins (G1, G2) and a nucleocaspid (N) protein [7,8]. The 5' end and 3' end of the three fragments are conserved, and are complementary in a reverse manner to form a panhandle structure. Previous studies determined that the genetic variability of *M* segments (envelope glycoproteins gene) is the most common in HV, which may be related to larger immune pressure given by the host infected with HV. Glycoprotein precursor (GPC) is an important structural protein with virulence loci, cell binding site, neutralizing epitopes, and type-specific antigen sites, and can stimulate the organism to produce neutralizing antibodies that have a protective effect in the infected animal and human body[9,10,11].

In Hebei Province, since the first HFRS case was reported in 1981, the disease has spread across the whole province and Hebei has become one of the most affected areas in China. Through the three epidemic peaks, the highest incidence rate of HFRS in Hebei Province was 7.6/10 million in 1999, and the cases have spread to 152 counties, an estimated 88% of all counties in Hebei Province. Although the epidemic situation of HFRS in Hebei Province is widely distributed and relatively concentrated, it has a dynamic change trend in the central regions (Shijiazhuang, Cangzhou, Hengshui, Baoding) and the northeast (Qinhuangdao, Tangshan) in recent years [12]. At present, the cases of northeast regions account for more than 80% of the total number of cases in Hebei Province, thus is main endemic area of HFRS in Hebei Province [13].

This study aimed to characterize the dominant genotype, analyze the *M* segment nucleotide acid sequence, clarify the geno-variation of HV harbored by rodents in main endemic areas of HFRS in Hebei Province of China over the time, and guide the prevention and control for this disease.

## Materials and Methods

### Ethics statement

This study was reviewed and approved by the Ethics Committee of the Hebei Province Centre for Disease Prevention and Control (IRB(P)2016-001). All animals were treated in strict according to the guidelines for the Monitoring Programs of HFRS from the Ministry of Health, China, under the protocols approved by the National Institute for Viral Disease Control and Prevention. This study did not involve endangered or protected species.

### Sample selection and origin

For this study, 19 rodent samples and 2 strains of HV were used. The rodents were collected in northeast Hebei Province (Qinhuangdao:39°83'–40°40'N, 118°87'–119°77'E; Tangshan: 39°27'–40°18'N, 117°73'–118°9'E) from 2004 to 2013. The rodents were placed on an 8-hour fast, anesthetized with sodium pentobarbital (65 mg/kg, i.p.). Lung tissues were flash frozen in liquid nitrogen and stored at −80°C until assayed. Lung tissues were isolated and the HV antigen was detected positive by indirect immunofluorescence. The 2 strains of HV were isolated from Qinhuangdao in 1993 and have been maintained in our laboratory. All the specimens were stored at −80°C. Each location involved in the study was permitted by Hebei Province Center for Disease Prevention and Control.

### Primers

A reverse transcription primer (*P14*) and two pairs of primers (*MP1*, *MP2* and *MP3*, *MP4*) for amplifying M segments were designed according to conserved sequences of *M* segments of HV from GenBank and corresponding references (Table 1).

**Table 1:** Primers for amplifying the M1 and M2 segments.

Primers	Location	Sequence (5’ →3’)	Meaning	Length (bp)
*P14*	1–14	TAGTAGTAGACTCC	+	reverse transcription
*MP1*	1–18	TAGTAGTAGACTCCGCAA	+	2353
*MP2*	2331–2353	TGGGCAATCTGGGGGGTTGCATG	−	
*MP3*	1936–1957	GTGGACTCTTCTTCTCATTATT	+	1716
*MP4*	3631–3651	TAGTAGTAGACTCCGCAAGAT	−	

### Extraction and preparation of total RNA

Total RNA was extracted from the rodent lung tissues and HV strains, and the procedures were performed in a laminar flow hood in a biosafety level 3 facility. The Maxwell 16 Tissue LEV Total RNA Purification Kit (Promega Corporation, USA) was used to extract RNA from 50 mg of lung tissues or 200 μl of HV strains in accordance with the manufacturer’s recommendations.

### Real time RT-PCR

The Hantavirus renal syndrome typing real time RT-PCR Kit (Shanghai ZJ Bio-Tech Co., Ltd., China) was used for detection and typing of HV. The reaction system included: 5 μl of total RNA as template, 18 μl of HFRS-I (HFRS-II) reaction mixture, 1 μl of enzyme for RT-PCR, and 1 μl of RNase free water. The real-time RT-PCR was performed as follows: initiation for 10 minutes at 45°C and 10 minutes at 95°C, followed by 40 cycles at 95°C for 15 seconds and 60°C for 1 minute.

### RT-PCR amplification

The RT reaction system included:10 μl of total RNA as template, 4 μl of 5× RT buffer, 2 μl of dNTP, 0.5 μl of AMV (Promega Corporation, USA), 0.5 μl of RNase, 2 μl of P14 and 1 μl of RNase free water. cDNA was synthesized after 42°C for 1 hour, 75°C for 15 minutes. The initial PCR reaction system was a 50 μl reaction mixture under the following conditions: 5 μl of cDNA as template, 5 μl of 10× LA Taq Buffer II, 5 μl of 25 mMol/L MgCl2, 6 μl of dNTP, 2 μl of MP1 and MP2 (MP3 and MP4) each, 0.5 μl of LA Taq (Takara Biotechnology Co., Ltd., Japan) 24.5 μl of ultrapure water. The initial PCR program for *M1* segments were amplified following a protocol that consisted of one cycle for 5 minutes at 94°C, and 35 cycles of denaturation at 94°C for 60 seconds, annealing at 60°C for 60 seconds, and extension at 72°C for 45 seconds. This was followed by a final extension at 72°C for 10 minutes. *M2* segments were amplified using the same conditions as *M1* except for annealing at 52°C for 60 seconds. The PCR products were analyzed by 0.8% agarose gel electrophoresis. The agarose Gel DNA Extraction Kit (Takara Biotechnology Co., Ltd., Japan) was used for purification of the objective gene fragments.

### T-A cloning

Using T-A cloning technique, the purified product (*M1* and *M2* segments) was cloned into pMD-19 vector (TaKaRa Biotechnology Co.,Ltd. Dalian, China), transformed into the *E*.*coli* competent cells DH5α, and the positive recombinant plasmids were screened by AMP/IPTG/X-gal, and restriction enzyme digestion and PCR identification.

### Nucleotide sequence determination and analysis

The positive recombinant plasmids identified were sequenced by Sangon Biotech (Shanghai) Corporation Ltd. The obtained nucleotide sequences were spliced to the complete *M* segments. Alignment and comparative nucleotide sequence analysis were carried out using DNASTAR7 (DNASTAR Inc. Madison, WI, USA).

### Phylogenetic analysis

Phylogenetic analysis was performed using MEGA 5.22. The sequences used in the phylogenetic analysis included sequences of HV isolates in the present study and other representative HV sequences from GenBank.

## Results

### Real time RT-PCR results

HV-positive rodent lungs and strains in northeast Hebei Province were analyzed with real time RT-PCR. The results indicated that all 21 positive specimens should be classified as Seoul virus (SEOV).

### Sequence analysis of *M* segment sequences

The recombinant plasmid of *M* segment was constructed and sequenced; the results showed that *M* segment sequence of all specimens were 3651 nt in length. The *M* segment consisted of 3 sections: 5’non-coding region (NCR), one open-reading frame (ORF), and 3’NCR. The nucleotide length of 5’NCR and 3’NCR was 47 nt and 203 nt, respectively. The *M* segment contained a single ORF (48 nt to 3448 nt) which encodes the M protein precursor of 1133 amino acids (aa). As a member of HV, all specimens had distinctive terminal complementary nucleotides that allow the folding of the viral genomic segments into “panhandle” hairpin structures.

We chose 11 typical specimens for sequencing; the results showed that there was high nucleotide homology, and both transition and transversion were found (Table 2). The nucleotide homology of these 11 specimens was 95.8%–99.8%, among which 93HBQ3 and 93HBQ4 was 95.8%–97.0% with other 9 specimens. Both 93HBQ3 and 93HBQ4 were 98.7%, and other 9 specimens were higher (98.6%–99.8%). Moreover, their nucleotide constitutions were similar with SEO type of HV strains (R22, L99, BjHD01, Z37); the nucleotide homology was 83.6%–99.2%. However, when compared with HTN type of HV strains (Q32, Lee, 76–118), the nucleotide homology was low (70.0%–71.7%) (Table 3). Results further showed that the variation of HV was small.

**Table 2:** HV-positive samples used in this study and real-time PCR types.

No.	Name	Host	Region	Time	IFA	Genotype	GenBank Accession No.
1	93HBQ3	R.n	Qinhuangdao	1993	+++	SEO	KM115585
2	93HBQ4	S.h	Qinhuangdao	1993	+++	SEO	KM115586
3	HBT20/2007	R.n	Tangshan	2007.3	+++	SEO	KM233656
4	HBQ43/2009	R.n	Qinhuangdao	2009.4	++	SEO	KM233657
5	HBT14/2012	R.n	Tangshan	2012.11	++++	SEO	KM233658
6	HBT3/2012	R.n	Tangshan	2012.10	+++	SEO	KM233661
7	HBQ5/2012	R.n	Qinhuangdao	2012.10	+++	SEO	KM233653
8	HBQ17/2012	M.m	Qinhuangdao	2012.11	+++	SEO	KM233655
9	HBQ7/2013	R.n	Qinhuangdao	2013.1	+++	SEO	KM233654
10	HBT49/2013	R.n	Tangshan	2013.3	++++	SEO	KM233659
11	HBT50/2013	R.n	Tangshan	2013.3	++++	SEO	KM233660

Note: R.n: *Rattus norvegicus;* S.h: *striped hamster;* M.m: *Mus musculus*.

**Table 3:** Comparison of the nucleotide and amino acid sequences of *M* segment (1–3651 nt).

	1	2	3	4	5	6	7	8	9	10	11	12	13	14	15	16	17	18	19
1 93HBQ3/KM115585	***	98.7	96.9	96.8	97.0	97.0	96.5	96.9	96.5	96.9	96.9	95.2	94.6	96.7	97.2	83.8	71.3	70.6	71.4
2 93HBQ4/KM115586	99.3	***	96.3	96.2	96.4	97.0	96.1	95.8	96.3	96.3	96.3	94.5	93.9	96.1	96.5	83.3	71.0	70.3	71.1
3 HBT20/2007/KM233656	99.0	99.0	***	99.2	99.3	99.4	99.0	98.7	99.1	99.1	99.1	95.5	94.9	99.0	97.3	84.1	71.1	71.1	71.7
4 HBQ43/2009/KM233657	98.9	98.9	99.6	***	99.2	99.3	98.9	98.8	99.1	99.0	99.1	95.5	95.0	99.0	97.2	83.8	70.8	70.8	71.4
5 HBT14/2012/KM233658	99.0	99.0	99.6	99.6	***	99.5	99.0	98.8	99.2	99.2	99.2	95.6	95.0	99.1	97.3	84.0	71.0	71.0	71.7
6 HBT3/2012/KM233661	99.0	99.0	99.6	99.6	99.6	***	99.1	98.9	99.3	98.9	99.2	95.6	95.1	99.2	97.5	84.2	71.0	71.1	71.7
7 HBQ5/2012/KM233653	99.2	99.2	99.8	99.7	99.8	99.8	***	98.5	99.2	98.7	98.9	95.5	94.9	98.8	97.2	83.8	71.1	71.0	71.6
8 HBQ17/2012/KM233655	98.4	98.3	98.9	98.9	98.9	98.9	99.1	***	98.7	99.2	98.7	95.2	94.7	98.6	97.0	83.6	70.7	70.6	71.3
9 HBQ7/2013/KM233654	99.2	99.2	99.8	99.7	99.8	99.8	100.0	99.1	***	99.8	99.2	95.6	95.0	99.0	97.3	84.0	71.0	70.9	71.6
10 HBT49/2013/KM233659	99.1	99.1	99.7	99.6	99.7	99.7	99.9	99.0	99.9	***	99.8	95.6	95.1	99.0	97.3	84.3	71.0	70.9	71.7
11 HBT50/2013/KM233660	99.1	99.1	99.7	99.6	99.7	99.7	99.9	99.0	99.9	99.8	***	95.7	95.1	98.9	97.3	84.2	71.0	70.9	71.7
12 L99/AF288298	98.2	98.2	98.7	98.6	98.7	98.7	98.9	98.0	98.9	98.8	98.8	***	99.1	95.3	95.7	84.1	70.7	71.0	71.2
13 R22/AF035834	95.0	95.0	95.4	95.3	95.4	95.4	95.6	94.7	95.6	95.5	95.5	95.7	***	94.8	95.2	83.6	70.3	70.6	70.7
14 BjHD01/DQ133505	99.0	99.0	99.6	99.6	99.6	99.6	99.8	98.9	99.8	99.7	99.7	98.7	95.5	***	97.1	83.9	71.0	70.9	71.7
15 Z37/AF187081	99.0	99.0	99.3	99.2	99.3	99.3	99.5	99.5	99.5	99.4	99.4	98.7	95.4	99.3	***	84.1	71.3	71.0	71.3
16 Gou3/AB027521	96.6	96.6	96.8	96.8	96.8	96.8	97.0	96.1	97.0	96.9	96.9	96.2	92.9	96.8	97.0	***	70.4	71.0	71.6
17 Q32/DQ371905	76.8	76.7	76.8	76.6	76.7	76.9	76.9	76.5	76.9	76.8	76.8	76.4	73.2	77.1	76.6	76.7	***	84.0	83.8
18 Lee/D00377	76.5	76.4	76.5	76.4	76.4	76.5	76.5	76.2	76.5	76.5	76.5	76.3	76.1	76.6	76.3	76.8	96.1	***	95.0
19 76-118/M14627	77.1	77.0	77.1	77.0	77.0	77.2	77.2	76.8	77.2	77.1	77.1	76.9	73.6	77.2	76.9	77.2	96.0	98.2	***

Note: Homology of the nucleotide sequences in upper triangle. Homology of amino acid sequences in lower triangle.

### Phylogenetic analysis of *M* segment sequences

**Fig 1:**
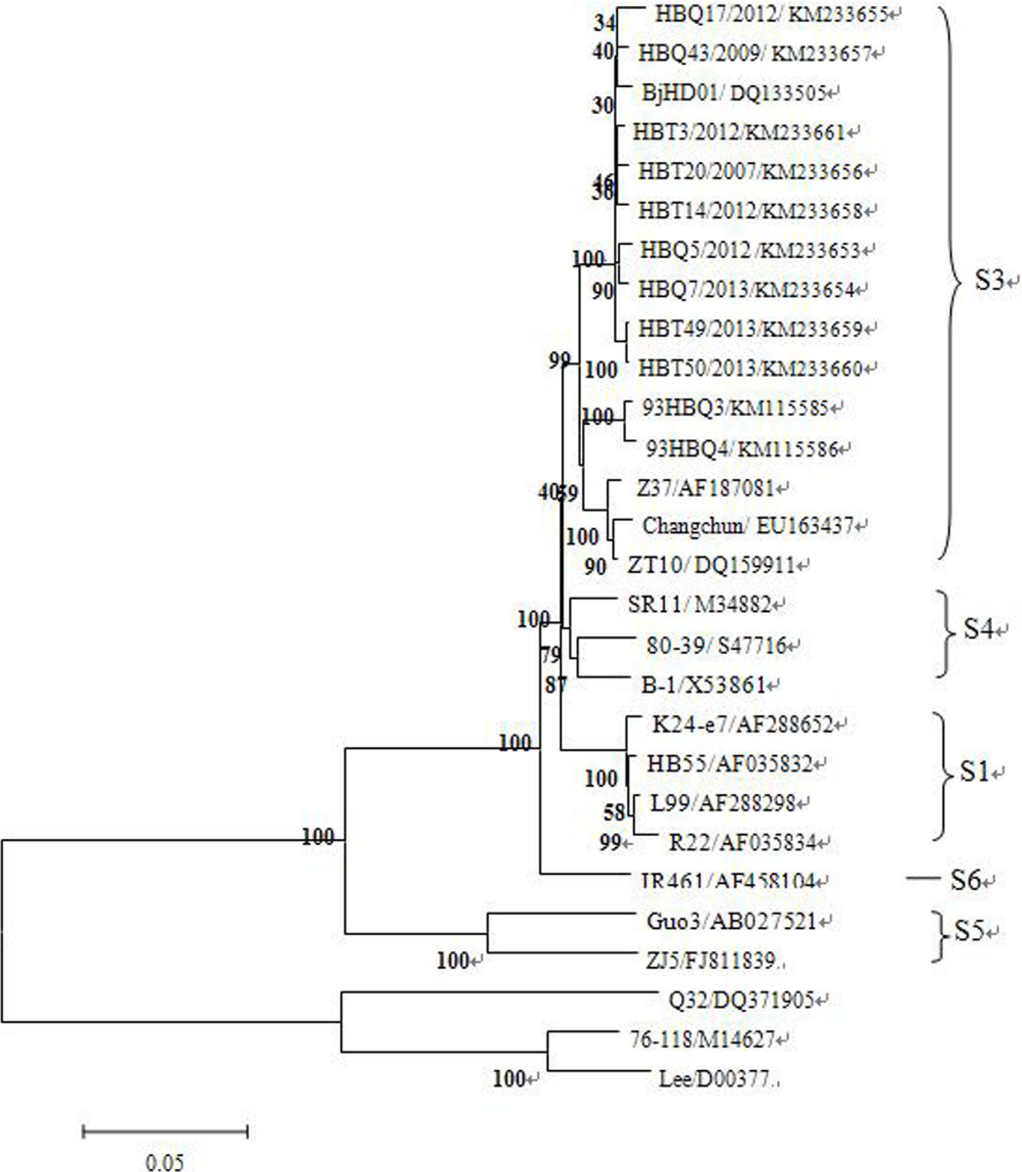
Phylogenetic trees for hantavirus based on the complete sequences of *M* segments (1-3651nt).

The phylogenetic tree of the *M* segment was constructed by MEGA package. The results showed that HBT-3/2012, HBT14/2012, HBT49/2013, HBT50/2013, HBQ20/2007, HBQ43/2009, HBQ5/2012, HBQ17/2012, HBQ7/2013 and BjHD01 had closer genetic relationship. Additionally, 93HBQ3, 93HBQ4 and Z37, ZT10 had closer genetic relationship. All 11 specimens and the reference strains were in the same clade, which belonged to the S3 subtype (Fig 1).

## Discussion

HFRS has been a serious public health concern in Hebei for many decades, as the incidence rate in Hebei Province is one of the highest in China. In recent years, HFRS cases were mainly concentrated in the northeast region of Hebei. In this study, 21 HV-positive specimens were amplified with real time RT-PCR from rodent lungs and strains in Hebei. We found that all 19 rodent lungs and 2 HV strains were SEO types of HV. Our results demonstrated that Hebei Province is a typical endemic area of SEOV at the genetic level, which is consistent with previous research studies[14].

HVs are enveloped, cytoplasmic viruses with a single-stranded, negative-sense RNA genome that consists of three segments. Viruses with genomes consisting of several segments have more chances for genetic variation. Previous studies have shown that the genetic variability of *M* segments is the most obvious of three segments in HV[15,16]. Thus we focused on the *M* segment as the classification and sequence analysis of HV.

Comparative nucleotide sequence analysis and phylogenetic analysis were performed using 11 sequences of complete *M* segments selected from 21 sequences of HV isolated in the present study together with standard strains of HV from GenBank. Sequence analysis of HV-positive clones showed that the nucleotide sequence homology of 11 strains shared 95.8%–99.8% amino acid sequence homology with each other and shared 98.4%–100% homology with standard strains of SEOV, which showed that the variation of HV in main endemic areas of HFRS in Hebei Province was low. This also indicated that was likely a relationship between subtype and geographic origin. Many studies considered that HV distributions showed geographical clustering within individual HV types, especially in SEOV[17,18]. In our study, we can conclude that the HV subtype is relatively conservative, with characteristics of regional stability. Phylogenetic analysis demonstrated that all strains are grouped into the same SEO subtype S3, but 93HBQ3 and 93HBQ4 stains isolated in 1993 were different from other strains isolated in recent years, thus HV had changed significantly in the long term. We can conclude that S3 was the major subtype in main endemic areas of HFRS in Hebei Province, and the genome sequence was relatively conserved for the homeotype of the *M* segment. However, the same subtype in different virus strains exhibits obvious variation with time, and may even create a new subtype.

In this study, we have analyzed the genotypes and genetic characteristics of HV in main endemic areas of HFRS in Hebei Province, which can provide further scientific evidence for prevention measures according to different genotypes of HV. Moreover, this study indicated that the variation of HV was low within the short term, but a significant change occurred over the long term, which provides reference for the research on gene variation and evolution of HV.

## Supporting Information

S1 Text Sequence information for M segments. (TXT)
